# Electrostatic Analysis of Bioactivity of Ti-6Al-4V Hydrophilic Surface with Laser Textured Micro-Square Convexes

**DOI:** 10.3390/ma15227942

**Published:** 2022-11-10

**Authors:** Si Li, Yangyang Jin, Shaoxian Bai, Jing Yang

**Affiliations:** 1 Zhejiang Provincial Key Laboratory of Silkworm Bioreactor and Biomedicine, College of Life Sciences, Zhejiang Sci-Tech University, Hangzhou 310018, China; 2 College of Mechanical Engineering, Zhejiang University of Technology, Hangzhou 310032, China

**Keywords:** electrostatic force, laser surface texturing, Ti-6Al-4V, wettability, bioactivity

## Abstract

At solid-liquid interfaces, charged particles within the electric double layer (EDL) are acted on by the electrostatic force, which may affect cell absorption and surface wettability. In this study, a model of the electrostatic force and surface tension of textured surfaces was presented. Then, the growth and adhesion of Murine osteoblasts (MC3T3-E1) cells on laser-ablated micro-square-textured Ti-6Al-4V surfaces were studied to demonstrate the use of a laser-processed texture to effectively improve bioactivity. Three different micro-square-textured hydrophilic surfaces, presenting lower contact angles of 19°, 22.5°, and 31.75° compared with that of a smooth surface (56.5°), were fabricated using a fiber-optic laser. Cellular morphology and initial cell attachment were analyzed by field emission scanning electron microscopy (SEM) and fluorescence microscopy, respectively. The results show that the electrostatic force not only made the textured surface more hydrophilic but also made the cells tend to adhere to the edges and corners of the protruding convexes. Cell morphology analysis also showed that cells would prefer to grow at the edges and corners of each micro-square convex protrusion. The laser-treated surfaces were more conducive to rapid cell growth and adhesion, and cells were preferentially attached on the hydrophilic-textured surfaces. Electrostatic force may be an important factor in effectively improving the bioactivity of Ti-6Al-4V surfaces, and the presence of more surface grooves would be more conducive to improving the bioactivity of cells.

## 1. Introduction

Excellent bioactivity has been always expected for titanium and its alloys used as bio-implants in clinical applications to increase adhesion with bone. Surface morphology has been proven to play an important role in promoting the spreading of mast cells, as well as cell migration and cell growth [1,2,3,4]. The enhancement of biological activity by surface geometric structure design and its mechanism have been much studied.

The use of laser surface-texturing, as a technique offering precise patterning with rapid speed and repeatability, has further deepened the understanding of surface texturing on biological activity in recent years [5,6,7,8,9]. The interaction of MC3T3 cells with laser-ablated micro-grooved titanium surfaces was shown by Soboyejo et al. [10]. The process of cell contact guidance was found to align with the grooves, in contrast to the random cell orientation with scar-tissue formation on a blast-textured surface [11,12]. Cell contact guidance was aligned along the direction of grooves and increased with decreasing longitudinal groove spacing. The adhesion strength was also found to increase on a laser-ablated surface [13].

More importantly, it has been found that there is a certain correlation between the biological activity of the textured surface and its wettability. An experimental study conducted by Kumari et al. [14] showed that the surface wettability of bare Ti-6Al-4V was enhanced after texturing with the contact angle falling from 89° to 71°−77°. Meanwhile, with texturing of the substrate, the percentage of adhered cells and average cell area were significantly improved. Other studies by Behera et al. [15] and Pratap [16] also found that surface texturing can effectively improve Ti-6Al-4V surface wettability. Since wettability reflects the movement of a liquid under microscopic forces at the interface, it may provide a potential way to study the bioactivity of textured surfaces from the perspective of interface electrostatic mechanics.

Charged particles are acted on by the electric field force of the double electric layer, referred to as the electrostatic force, which makes the particles tend to approach the solid-liquid interface [17,18]. In aqueous solution, protein particles and cell surfaces often exhibit charged characteristics. Theoretically, the electrostatic force makes cells and proteins move toward the solid interface [19]. However, it has been reported that the action of the double layer on the charged particles significantly affects the bulk viscosity and motion behavior of the fluid [19,20,21,22]. Theoretically, the electrostatic force will also affect the wetting characteristics of the liquid at the interface. This may mean that the electrostatic force will affect both cell bioactivity and wettability.

Recently published works show that surface charge exerts a significant influence on the delivery of biochemical agents, such as genes, proteins, RNA molecules [23], and downstream molecular signaling cascades [24], and also enhances cellular osteogenic differentiation [25]. An experimental investigation and molecular dynamics simulation by Tang et al. [26] confirmed the surface potential-induced conformational change in adsorbed proteins. In addition to chemical gradients and gene regulatory networks, endogenous ion flows are key regulators of cell behavior [27]. A cell behavior analysis by Zhang et al. [28] showed that the increase in charge density was conducive to promoting cell adhesion and the formation of filopodia, while the nonuniform spatial distribution of charge promoted an oriented arrangement of cells; both features accelerated cell migration.

In this work, EDL effects on cell adsorption and wettability were analyzed for textured surfaces in terms of EDL adsorption force and surface tension. In addition, the growth and adhesion of MC3T3-E1 cells on laser micro-square-convexed Ti-6Al-4V surfaces were studied, to analyze the influence of the electrostatic force on bioactivity.

## 2. Electrostatic Analysis Model

**Figure 1:**
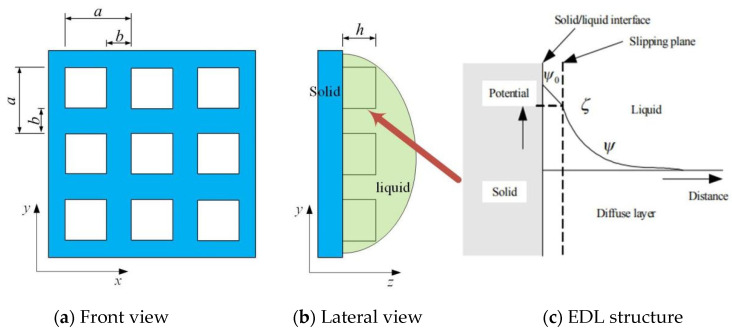
Schematic view of protuberant texture surface and EDL structure: (**a**) front view of surface texture; (**b**) lateral view of surface texture; (**c**) EDL structure at interface of solid and liquid.

The schematic view of the laser-ablated convex textured surface adopted in this study is shown in Figure 1. Here, a texture consisting of micro-square convexes (blocks) is formed by laser-processed transverse and longitudinal micro-grooves cut into the surface. The clearances between grooves in both transverse and longitudinal directions were the same (*a* − *b*). The width of the micro-grooves in both transverse and longitudinal directions was also the same (*b*), and their depth was set as *h*. It has been shown that a convex texture can make the surface more hydrophilic [29,30].

It is well known that most solid surfaces carry electrostatic charges, i.e., an electrical surface potential. If the liquid contains a certain number of ions, the electrostatic charges on the solid surfaces will attract the counterions in the liquid. The rearrangement of the charges on the solid surface and the balancing charges in the liquid is called the electrical double layer, as illustrated in Figure 1.

The electrical potential at the solid-liquid interface *ψ*_0_ is difficult to measure directly. However, the electrical potential at the slipping plane, called the zeta potential *ζ* can be measured experimentally. According to the Debye–Hückel approximation [17,18], the electrical potential *ψ*_0_ at the solid-liquid interface is close to the value of the zeta potential *ζ.* By solving the Poisson–Boltzmann equation with appropriate boundary conditions, the electrical potential distribution *ψ* of the EDL can be obtained.

In the micro-environment of cell growth, the charge characteristics of protein molecules make their concentration higher at the interface than that of other parts under the action of the electrostatic force, which will be conducive to promoting cell growth.

### 2.1. EDL Adsorption Force

According to the theory of electrostatics, the relationship between the electrical potential *ψ* and local net charge density per unit volume *ρ*_e_ at any point in the solution is described by the Poisson equation [31]:

(1) $$\nabla^{2}\psi =-\frac{4\pi \rho_{e}}{\varepsilon }$$

where *ε* is the absolute dielectric constant of the fluid.

The net volume charge density *ρ*_e_ is proportional to the concentration difference between symmetric cations and anions:

(2) $$\rho_{e}=-2n_{0}z_{e}e\sinh\frac{z_{e}e\psi }{k_{b}T}$$

where *n*_0_ is the number concentration, *z*_e_ is the electrovalence of the ion, *e* is the elementary protonic charge, *k*_b_ is the Boltzmann constant, and *T* is the temperature.

In a textured surface solid-liquid system, as shown in Figure 1, there is one EDL at each interface between the solid and liquid. For the solid-liquid interface at the groove bottom in the depth direction *z*, the electrical potential distribution *ψ*_z_ can be expressed as follows:

(3) $$\psi_{z}=\zeta \exp(-kz)$$

The local charge density distribution can be expressed as:

(4) $$\rho_{e,z}=-\frac{k^{2}\varepsilon \zeta }{4\pi }\sinh\frac{z_{e}e\psi_{z}}{k_{b}T}$$

Here, *k* is the Debye reciprocal length parameter:

(5) $$k^{2}=\frac{8\pi n_{0}e^{2}z_{e}^{2}}{\varepsilon k_{b}T}$$

Generally, *k*^−1^ refers to the characteristic thickness of the EDL and is a function of the electrolyte concentration.

When the clearance between micro-square-convexes *b* is very small, the two EDLs are spliced. So, in the clearance between two micro-square-convexes, the electrical potential distribution *ψ_i_* is as follows:

(6) $$\psi_{i}=\frac{4kT}{z_{e}e}\gamma \exp(-kx)+\frac{4kT}{z_{e}e}\gamma \exp(k(x-b))$$

where, *i* = *x*, *y*.

Then, the local charge density distribution can be expressed as:

(7) $$\rho_{e,i}=-\frac{k^{2}\varepsilon \zeta }{4\pi }\sinh\frac{z_{e}e\psi_{i}}{k_{b}T}$$

Proteins and other particles needed for cell growth are often charged in aqueous solution. Meanwhile, the cell surface also exhibits certain charged properties. The charged particles in solution tend to move towards the interface attracted by electrostatic forces induced by the EDL.

For a smooth surface, the electrostatic force, also called the EDL adsorption force, can be obtained as:

(8) $$p_{\mathrm{edl},z}^{}=\frac{\partial \psi_{z}}{\partial z}\rho_{e,z}^{}$$

So, at the clearance edge, the EDL adsorption force is:

(9) $$p_{\mathrm{edl},i}^{}=\sqrt{{\left(\frac{\partial \psi_{i}}{\partial i}\right)}^{2}\rho_{e,i}^{2}+{\left(\frac{\partial \psi_{z}}{\partial z}\right)}^{2}\rho_{e,z}^{2}}$$

For the bottom corners, the EDL adsorption force may be expressed as:

(10) $$p_{\mathrm{edl},\mathrm{tri}-\mathrm{corner}}^{}=\sqrt{3}\frac{\partial \psi_{z}}{\partial z}\rho_{e,z}^{}$$

### 2.2. EDL Surface Tension

The electrostatic force forms additional pressure at the solid-liquid interface, which affects the infiltration and spreading of the liquid.

For smooth surfaces, the equivalent surface tension generated by electrostatic forces can be calculated as:

(11) $$\gamma_{\mathrm{edl},0}=\frac{{\int_{0}^{a-b}p_{\mathrm{edl}}dy|}_{z=0}}{a-b}$$

In the same way, at the top of the clearance, the equivalent surface tension generated by electrostatic forces can be calculated as:

(12) $$\gamma_{\mathrm{edl},h}=\frac{{\int_{0}^{b}p_{\mathrm{edl}}dy|}_{z=h}}{b}$$

Based on the Young equation of an isotropic, homogeneous, and smooth ideal surface, the relation between the intrinsic contact angle and the surface tension of the solid surface is given as:

(13) $$\gamma_{\mathrm{sv}}=\gamma_{\mathrm{sl}}+\gamma_{\mathrm{lv}}\cos\;\theta$$

where, *γ*_sv_, *γ*_lv_, *γ*_sl_ are the surface tension of the solid surface, liquid surface, and solid-liquid interface, respectively; and *θ* is the inherent contact angle on the solid surface. When the electrostatic force is considered, Equation (13) can be modified as:

(14) $$\gamma_{\mathrm{sv}}=\gamma_{\mathrm{sl}}+\left(\gamma_{\mathrm{lv}}-\gamma_{\mathrm{edl},0}-\frac{b}{a-b}\gamma_{\mathrm{edl},h}\right)\cos\;\theta$$

## 3. Experimental Section

### 3.1. Preparation of Textured Samples

The micro-square-convex morphology was cut onto the Ti-6Al-4V surface using an optical-fiber laser marking machine (Han’s Laser, Shenzhen, China). The different morphological parameters were obtained by changing the laser processing parameters, including the laser output power and the number of processing units.

**Figure 2:**
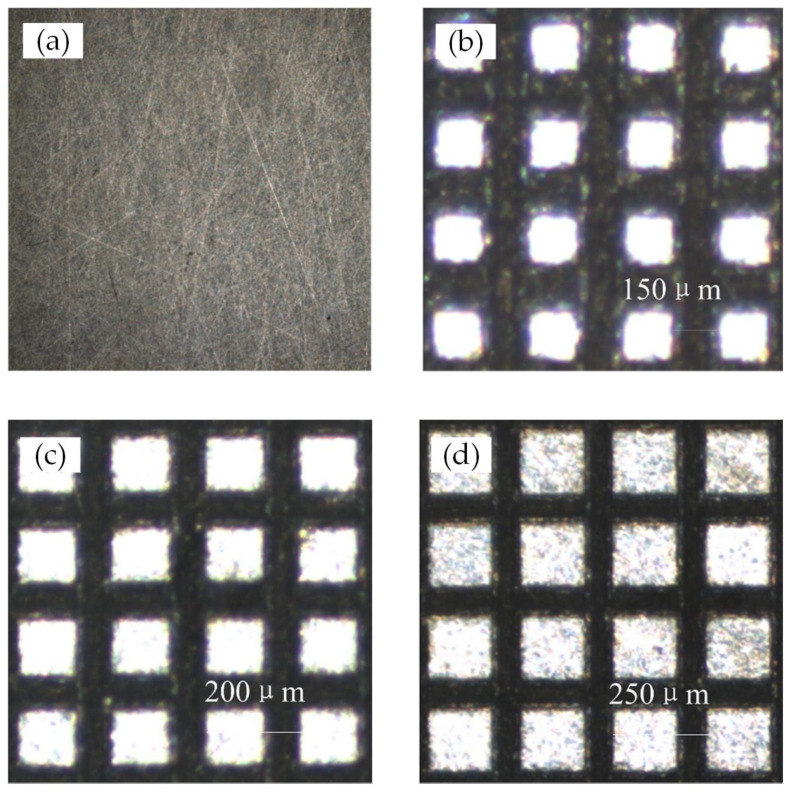
2D profiles for different types of untreated or laser-treated surface: (**a**) smooth surface; (**b**) Textured surface 1; (**c**) Textured surface 2; and (**d**) Textured surface 3.

After processing, the sample surfaces were polished, and the roughness of the non-texture area was measured and found to be *R*a = 0.1 μm. Then, the Ti-6Al-4V samples were cleaned in an ultrasonic cleaner with hydrochloric acid, acetone, alcohol, and deionized water. The morphological parameters measured using a surface profilometer are listed in Table 1, and the 2D profiles of the smooth and textured surfaces are shown in Figure 2.

**Figure 3:**
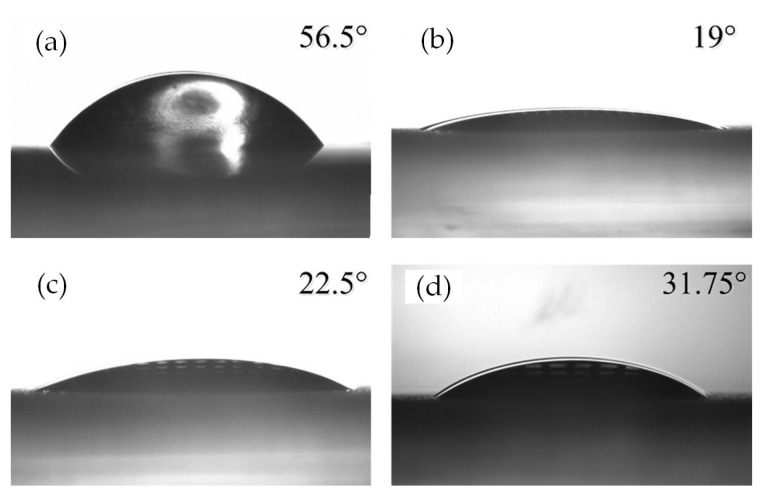
Static contact angle of different surfaces: (**a**) smooth surface; (**b**) Textured surface 1; (**c**) Textured surface 2; and (**d**) Textured surface 3.

In addition, to demonstrate surface energy and wettability, the static contact angle of cells culture fluid on both the smooth and textured surfaces was measured by the sessile drop method. The volume of the liquid was 2 μL, and the results are shown in Figure 3.

In order to analyze the EDL influence on wettability, contact angles of the above surfaces were calculated by the present model described in Section 2.2. The values of the contact angles are listed in Table 2. Here, the liquid-gas interface tension is *γ*_lv_ = 71.42 × 10^−3^ N/m, number concentration *n*_0_ = 10^−7^ mol/L, electrovalence of ion *z*_e_ = 1, and elementary protonic charge *e* = 1.60 × 10^−19^ C [17,18].

It can be seen from Table 2, that, when the Zeta potential *ζ* = 9.26 mV, the theoretical values of the contact angle agreed well with the measured values. This means that the EDL electrostatic force was an important factor affecting the wettability. Meanwhile, the EDL made the surface more hydrophilic.

It can also be seen that difference between the values of the calculated and experimental angles was up to 42%. In this case, the theoretical contact angle for texture 1 was 1.34 times greater than the measured value, and for texture 2 the experimental value was 1.42 times less than the theoretical one. The reason for this was that the CA may be also affected by other factors such as droplet size [32] and chemical elements [33].

**Figure 4:**
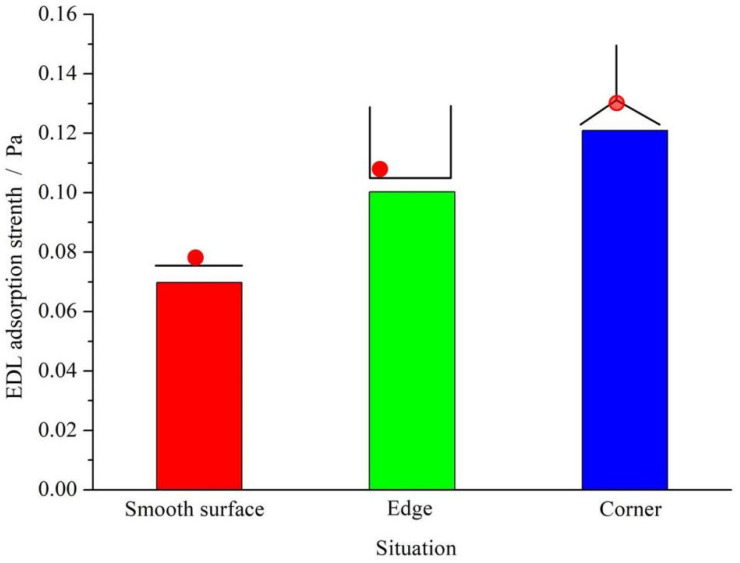
EDL adsorption forces (electrostatic force) at different surface positions.

Further, the EDL adsorption forces were calculated at the textured surfaces as shown in Figure 4. Obviously, the EDL adsorption force (electrostatic force) at the edges and corners was much higher than that at the smooth surface. So, it may be predicted that the cell prefers to grow along the edges and corners because of a higher EDL adsorption force.

### 3.2. Bioactivity of Ti-6Al-4V (Laser-Treated) Surface Assay

#### 3.2.1. Materials

Murine osteoblasts (MC3T3-E1) (provided by the Institute of Biochemistry of Zhejiang Sci-Tech University) were used to study the growth and adhesion on the laser-treated surfaces. The cells were cultured in MEM Alpha Modification (1×) (MEM-α, HyClone) with added 10% Fetal Bovine Serum (FBS, HyClone) and 1% penicillin/streptomycin (Gibco) at 37 °C in a humidified 5% CO_2_ atmosphere. Before the cell assay, the samples were cleaned with an ultrasonic cleaner for 30 min, then soaked in 2% hydrochloric acid overnight after being washed several times with ddH_2_O. The samples were thoroughly sterilized in an autoclave at 121 °C for 30 min. Cell numbers were counted with a C-Chip Automated Cell Counter (NanoEnTeK, Seoul, Korea).

#### 3.2.2. Cell Culture

MC3T3-E1 cells were digested using trypsin (Gibco) with ethylenediaminetetraacetic acid (EDTA) for 1 min to make the adherent cells into a cell suspension, and then the cell suspension was transferred to a 15 mL centrifuge tube. After centrifuging at 1000 rpm for 5 min, the 1-mL cell suspension was diluted 5 times with cell culture medium and placed in a counting cell, and transferred to the C-Chip. The cell suspension was diluted to the concentration of 2.5 × 10^4^ cells mL^−1^ according to the cell count results. There were three types of samples (labeled Texture 1, Texture 2, and Texture 3) whose surfaces had been laser-treated. The untreated samples (labeled Smooth) were used as a negative control. Six samples, each, of the Smooth, Texture 1, Texture 2, and Texture 3 surfaces, a total of 24 samples, were put into 24 wells. Then, to each of these 24 wells was added 500 µL of the cell suspension. A total of three 48-well cell culture plates were used and left to culture for 3 h, 6 h, and 22 h, respectively.

#### 3.2.3. Field Emission Scanning Electron Microscopy (SEM) Analysis Sample Preparation

After the MC3T3-E1 cells were cultured for 3 h, 6 h, or 22 h, the culture medium was carefully pipetted out, and then rinsed with PBS buffer. The cells on the samples were fixed with 2.5% glutaraldehyde for 30 min and cleaned three times with PBS buffer. Afterward, each sample underwent graded dehydration with 60%, 70%, 80%, 90%, and 100% ethyl alcohol for 30 min, respectively. Then the samples were dried at room temperature before, finally, being sputter-coated with gold for SEM analysis(Phenom, Netherlands).

#### 3.2.4. Fluorescence Staining Experiment

The method of cell culture was as above. Then the fluorescence staining experiment was conducted with propidium iodide (PI) for the cells cultured for 3 h, 6 h, or 22 h. After rinsing the surface of the samples with PBS buffer, cells adhered on the surface of the samples were fixed for 10 min with 95% ethanol. Afterward, to every well was added 200 µL of cell dye buffer and 5 µL of PI, and the cells were stained at 4 °C in the dark. After cleaning with PBS buffer for 30 min and drying at room temperature, the cells were observed with a stereo fluorescence microscope (NIKON SMZ25, Japan) and pictures were taken at x15.75 magnification for three randomly-selected areas per well. Then the cell numbers were manually counted with NIS-Elements (Nikon, Japan) according to these pictures. The cell numbers of each well were taken as the average number of the counts from the three pictures. Finally, the average was taken of the cell number from the six repeated experiments for each surface.

## 4. Results and Discussion

### 4.1. Cellular Morphology Analysis

**Figure 5:**
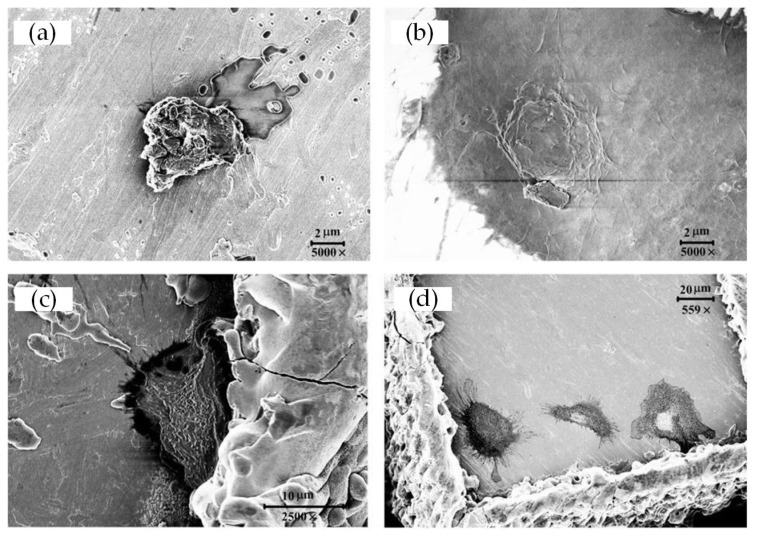
SEM picture of MC3T3-E1 cells morphology grown on the surface of samples (3 h): (**a**) Smooth; (**b**) Texture 1; (**c**) Texture 2; and (**d**) Texture 3.

**Figure 6:**
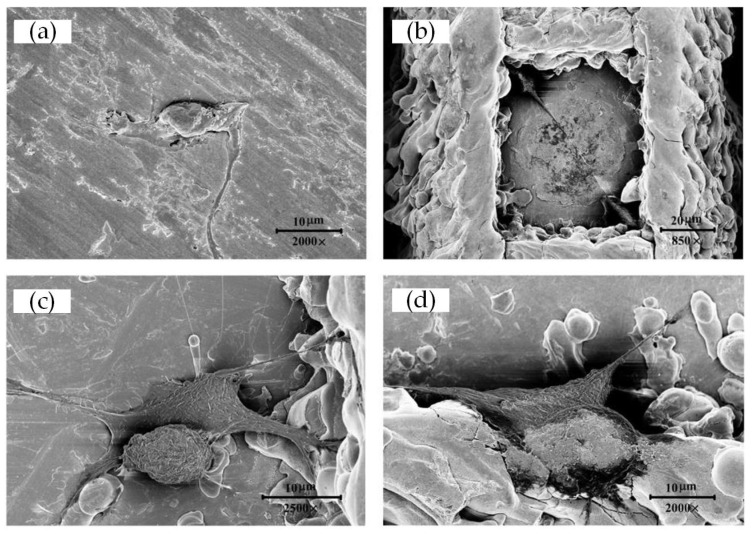
SEM picture of MC3T3-E1 cells morphology grown on the surface of samples (22 h): (**a**) Smooth; (**b**) Texture 1; (**c**) Texture 2; and (**d**) Texture 3.

Cell morphology after 3 h and 22 h of growth was visualized via SEM to reveal the initial cell adhesion and interactions with the substrates (Figure 5 and Figure 6). As is shown in Figure 5 and Figure 6, cells adhered on the surfaces of both the treated and untreated samples. The difference is that cells cultured for 3 h showed a spherical shape, while cells cultured for 22 h were extensively spread over the surface showing polygonal shapes or spindle shapes. In addition, cells cultured for 22 h extended significantly more, and longer, pseudopodia than cells cultured for only 3 h to help them adhere on the surface of the samples, exchange nutrients, and transduce signals between cells.

As predicted in Section 3.1, on the surface of laser-treated samples, cells preferred to grow at the edges and corners of each micro-square convex with their pseudopodia helping them achieve better adhesion; with a large number of cells amplifying to the surroundings as time went by. However, at the same time, cells were randomly distributed, with a large number of the population spreading out extensively on the untreated samples whose surfaces were relatively smooth.

In addition, as shown in Figure 5, cells cultured for 3 h exhibited a spherical shape that didn’t adhere closely to the surface of the non-treated samples. In contrast, on the laser-treated samples, the cells cultured for 3 h presented a spiny state stretching out a large number of short pseudopodia to help them quickly adhere to the surface.

### 4.2. Initial Cell Attachment and Viability

**Figure 7:**
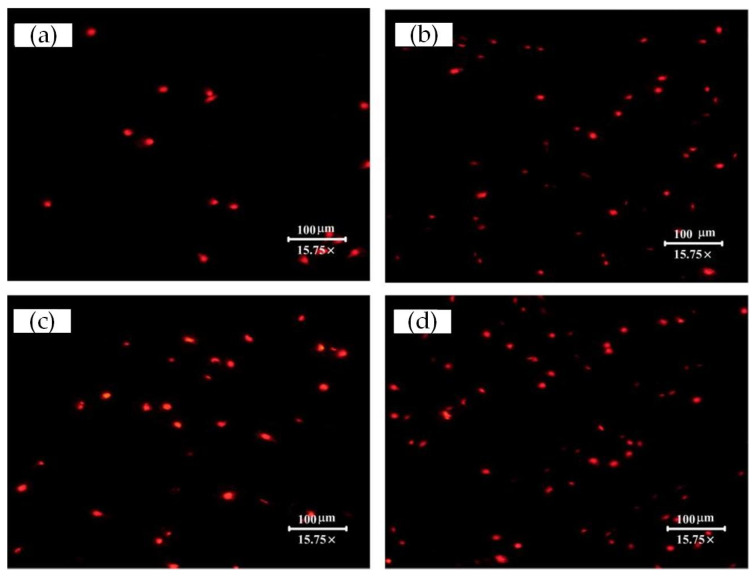
Stereo fluorescence microscope pictures taken for MC3T3-E1 cells adherent on the different surfaces after culturing for 3 h presented the variation in cell density and cell numbers: (**a**) Smooth; (**b**) Texture 1; (**c**) Texture 2; and (**d**) Texture 3.

**Figure 8:**
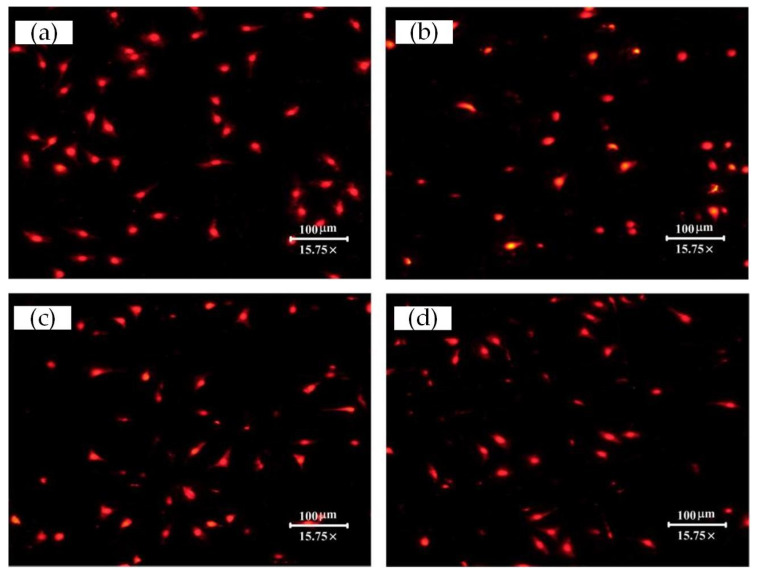
Stereo fluorescence microscope pictures taken for MC3T3-E1 cells adherent on the different surfaces after culturing for 6 h presented the variation in cell density and cell numbers: (**a**) Smooth; (**b**)Texture 1; (**c**)Texture 2; and (**d**)Texture 3.

**Figure 9:**
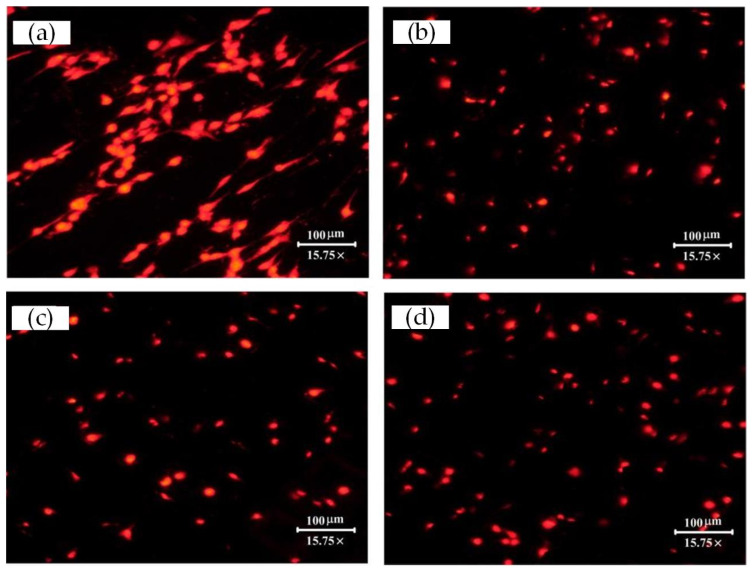
Stereo fluorescence microscope pictures taken for MC3T3-E1 cells adherent on the different surfaces after culturing for 22 h presented the variation in cell density and cell numbers: (**a**) Smooth; (**b**) Texture 1; (**c**) Texture 2; and (**d**) Texture 3.

Fluorescence microscopy pictures of cells cultured for 3 h, 6 h, or 22 h are presented in Figure 7, Figure 8 and Figure 9, respectively. Semi-quantification on the number/density of the staining cell results (Figure 7) indicated that all the surface treatment samples were capable of supporting the initial attachment of MC3T3-E1 cells within 3 h of incubation. Any one of three types of laser-treated surfaces of Texture, Texture 2, and Texture 3 have significantly higher numbers of attached cells than the untreated samples (Smooth: 27 ± 15, Texture 1: 59 ± 7, Texture 2: 55 ± 6, Texture 3: 60 ± 4; mean ± SD). This result shows that the laser-treated surfaces are more conducive to rapid cell growth and adhesion, which is consistent with the result of the SEM analysis. According to the staining cell results (Figure 8), the number/density of MC3T3-E1 cells had no obvious difference between treated and untreated samples within 6 h of incubation (Smooth: 53 ± 3, Texture 1: 55 ± 16, Texture 2: 63 ± 3, Texture 3: 59 ± 11; mean ± SD). However, the results of culturing for 22 h (Figure 9, Smooth: 67 ± 32, Texture 1: 88 ± 37, Texture 2: 65 ± 6, Texture 3: 66 ± 12; mean ± SD) indicated that the number/density of the laser-treated samples of Texture 2 and Texture 3 was similar to that of the untreated samples, but the samples of Texture 1 were significantly greater. The whole cell number and density are increasing with the extension of incubation time from 3 h to 22 h. This phenomenon showed that MC3T3-E1 cells can grow and reproduce on the surface of Ti-6Al-4V whether treated or not. A small number of both dead and disrupted cells suggested high viability of cells on the laser-treated and untreated surfaces.

It should be noted that, compared with the smooth surface, the cell size seems different on the surface of the Texture 1, Texture 2, and Texture 3 surfaces, especially after 22 h of culturing (Figure 9). The reason for this is that, for the textured surfaces, the fluorescence microscope needs to focus at some position between the groove top and bottom to shoot the cells on both the top and bottom surfaces. This resulted in the cells presenting as both bright and dim points. But, for the smooth surface, the fluorescence microscope needs only to focus on one point of the surface to shoot the cells, so the cells present as clearer, larger and brighter points in the images.

### 4.3. Difference Analysis

**Figure 10:**
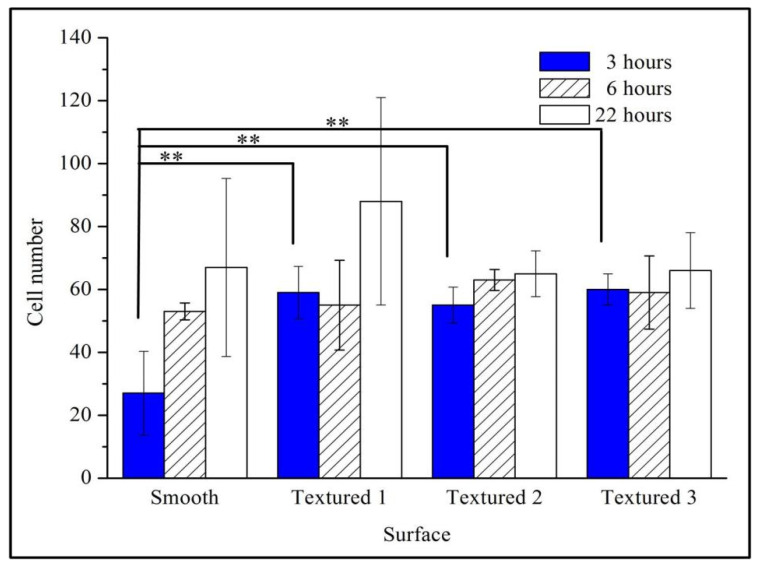
The final results of MC3T3-E1 cell numbers after 3 h, 6 h, and 22 h of growth. Smooth; Texture 1; Texture 2; and Texture 3. ** *p* < 0.01, versus smooth surface.

Figure 10 gives the results of the three time points in the repeated experiments. The number of cells depended on the average number of six samples, each, of Smooth, Texture 1, Texture 2, and Texture 3 at the three time points in each repeated experiment. Because the number of cells needs to be counted manually, it is easy for a large deviation to develop, so, analyses of variance were used to determine the significance of the observed differences. A *p*-value < 0.05 was considered statistically significant and < 0.01 was considered very significant.

According to the final results (Figure 10) of cell numbers after 22 h of growth, the sample of Texture 1 had obvious advantages. Therefore, the surface of the sample Texture 1 was the most suitable for MC3T3-E1 cells adhesion and growth, making it the ideal biomedical material.

According to the present model, the more grooves there are, the more conducive the textured surface is to the aggregation of protein particles, which is more conducive to promoting cell growth. In the experiment, the sample of Texture 1 had the most grooves, so the cell activity was the best with this sample. It may be concluded that more grooves are more conducive to improving the bioactivity of cells.

## 5. Conclusions

The study presents a model of electrostatic adsorption based on the electric double layer, and then the growth and adhesion of MC3T3-E1 cells on laser micro-square-convexed Ti-6Al-4V surfaces were investigated. Cellular morphology and initial cell attachment were analyzed. The following conclusions may be drawn:

(1) The electrostatic force not only make the textured surface more hydrophilic but also make the cells tend to adhere to the edges and corners of any protrusion. Cells would prefer to grow in the edges and corners of each micro-square convexes.

(2) Electrostatic force may be an important factor in effectively improving the bioactivity of Ti-6Al-4V surfaces. The addition of more surface grooves would be more conducive to improving the bioactivity of cells.

## Figures and Tables

**Table 1:** Detailed morphological parameters of the micro-square-convex surfaces.

Morphology	Serial Number	*a*/μm	*b*/μm	*h*/μm
Smooth surface	Smooth	/		
Micro-square-convex surface	Texture 1	110	40	5
	Texture 2	160	40	5
	Texture 3	210	40	5

**Table 2:** Prediction of contact angle (CA) on micro-square-convex surfaces.

Serial Number	*z* /mV	*γ*_edl,0_ /N/m	*γ*_edl,h_ /N/m	CA Experimental Value *θ*/°	CA Theoretical Value *θ*/°
Smooth	9.26	0.0697	/	56.5	/
Texture 1			0.015	19	14.1
Texture 2				22.5	32.0
Texture 3				31.75	37.2

## Data Availability

Not applicable.
